# “Nothing for Us Without Us”: An Evaluation of Patient Engagement in an HIV Care Improvement Collaborative in the Caribbean

**DOI:** 10.9745/GHSP-D-21-00390

**Published:** 2022-06-29

**Authors:** Shay Bluemer-Miroite, Katy Potter, Elizabeth Blanton, Georgia Simmonds, Conrad Mitchell, Kenyatta Barnaby, Karen Askov Zeribi, Dale Babb, Nicola Skyers, Gabrielle O'Malley, Clive Anderson

**Affiliations:** a Shift, Seattle, WA, USA.; b Independent consultant, Seattle, WA, USA.; c University of Washington, Seattle, WA, USA.; d Caribbean Training and Education Center for Health, Kingston, Jamaica.; e International Training and Education Center for Health, Port of Spain, Trinidad and Tobago.; f Ladymeade Reference Unit, Barbados Ministry of Health, St. Michael, Barbados.; g HIV Programme, Jamaica Ministry of Health, Kingston, Jamaica.; h International Training and Education Center for Health, Seattle, WA, USA.; i International Training and Education Center for Health, Kingston, Jamaica.

## Abstract

**Introduction:**

Patient engagement is increasingly recognized as a key strategy to promote patient-centered care and accelerate health care improvements. Ensuring patient participation in improvement efforts is particularly important with stigmatized illnesses and marginalized populations. Despite the attention it has garnered, patient engagement is still not widely implemented and has not been well documented in global health literature.

**Methods:**

We implemented a patient-engagement strategy to involve people living with HIV in quality improvement efforts. As part of the Caribbean Regional Quality Improvement Collaborative, quality improvement teams from Barbados (1 team), Jamaica (20 teams), Suriname (3 teams), and Trinidad and Tobago (2 teams) engaged health care providers from care facilities and people living with HIV to serve as community representatives (CRs) to lead the improvement efforts alongside them. This strategy was evaluated via a mixed method design that included 2 rounds of semistructured, in-depth interviews with patients and providers.

**Results:**

Findings suggest that the patient engagement strategy had several key strengths: it promoted the collection, use, and appreciation of patient input to inform health care improvements at the facility level; facilitated the empowerment of CRs; enhanced mutual understanding and empathy between CRs and providers; and helped to dispel HIV stigma and discrimination in health care settings. Moreover, both health care providers and CRs reported that CR opinions and perspectives are as important as providers' and that CR participation in the improvement process was beneficial.

## Abstract (precis)

This evaluation suggests that it is both possible and valuable to include patients as partners in quality improvement efforts, especially when resources must be prioritized for the highest impact efforts. Patient engagement in the improvement process is particularly powerful when addressing illnesses that may be stigmatized such as HIV.

## Teaser

**Key Findings:**

- Intentionally building pathways for patient partnership in the improvement collaborative resulted in increased integration of and appreciation for patient perspectives.
- The conditions created in the Caribbean Regional Quality Improvement Collaborative resulted in increased mutual understanding and empathy between health care providers and people living with HIV, both of whom believed it benefited the quality improvement work.

**Key Implications:**

- In addressing the challenges of improving quality of care and improving health outcomes in lower-resource countries, policy makers should leverage quality improvement initiatives to integrate patients and those who work in the system to identify impactful changes.
- Program managers should intentionally design for authentic engagement with patients in improvement initiatives including creating optimal conditions of intergroup contact theory: equal group status within the intergroup setting, common goals, cooperation towards goals, and institutional support.

## INTRODUCTION

Patient engagement is increasingly recognized as a key strategy to promote patient-centered care and accelerate health care improvements.^1^^–^^4^ It can be broadly defined as the involvement of patients, their families, or representatives working in active partnership with health professionals to improve care.^5^ Patients may participate at many levels of the health care system, from individual care and organizational improvement to system design, policy making, monitoring, evaluation, and research.^5^^,^^6^ Benefits of patient engagement include enhanced patient activation; more effective patient-provider interactions; increased client satisfaction; reduced costs; and improved patient safety, quality of care, and health outcomes.^7^^–^^13^

While patient engagement has garnered significant attention, it is still not widely implemented. One fundamental issue is that the concept of “patients as partners” is not ingrained in the culture of care.^1^^,^^14^ Established power differentials in the health system can make it difficult for patients' voices to be heard and for their expertise via lived experience to be equally valued.^14^^,^^15^ There is also a great deal of uncertainty about how to meaningfully involve patients as interventions tend to employ them in more consultative roles rather than truly collaborative ones.^6^^,^^16^ Furthermore, marginalized groups tend to be especially underrepresented in patient engagement activities, despite their often-increased risk of experiencing health problems.^17^^–^^19^

Most patient engagement interventions have focused on improving health at the individual-level, rather than addressing broader systems issues.^20^^–^^22^ Quality improvement (QI) initiatives aim to strengthen health care quality by focusing on system failures and direct efforts at redesign by engaging a broad array of stakeholders.^23^ While the critical role of patient engagement in QI in high-income countries is well documented, evidence in low- and middle-income countries is remarkably scant.^24^ Given the successful application of QI to improve health systems around the world,^25^ omitting patient voices from QI efforts is a missed opportunity.^26^

Most patient engagement interventions have focused on improving health at the individual-level, rather than addressing broader systems issues.

We describe strategies to partner with patients in the Caribbean to improve HIV care and treatment within a “QI collaborative”^27^—health care facilities working together over a discrete period to improve a specific outcome or aim. Facilities and health care providers (HCPs) in the Caribbean rarely involve patients as equal members of a health care team, and stigma and discrimination against people living with HIV (PLHIV) remain a significant barrier to care.^28^^–^^30^ For some clients, this barrier is exacerbated by stigma and discrimination against key populations such as men who have sex with men (MSM), commercial sex workers (CSW), and transgender persons.^31^^–^^33^ Within this context, a patient engagement strategy was developed to create systems for collection and integration of PLHIV feedback; ensure QI teams test changes that align with PLHIV priorities; create mechanisms for PLHIV leadership and recruitment; decrease HIV-related stigma and discrimination in health care settings; and ultimately, improve health outcomes among PLHIV in the Caribbean region.

## CARIBBEAN REGIONAL QUALITY IMPROVEMENT COLLABORATIVE INTERVENTION

In 2015, The International Training and Education Center for Health (I-TECH) at the University of Washington initiated the Caribbean Regional QI Collaborative (CaReQIC), which supports a team-based approach to addressing barriers to high-quality health care. Foundational to this approach are rapid, small-scale tests of change in clinic processes, referred to as plan-do-study-act (PDSA) cycles, to achieve a predetermined, measurable aim. PDSA cycles facilitate the adaptation of interventions that have worked elsewhere to a local context. As teams test changes to their existing systems, they adapt and assess the effectiveness of those changes. Teams then implement those that have the greatest positive impact on their system, thus building iteratively toward their longer-term aim. Facility-based teams are brought together (virtually or in person) for periodic learning sessions where they are taught QI methods, share results of their QI work, and learn from and motivate each other. Between learning sessions, teams receive coaching from local, regional, and international experts, participate in webinars, test changes using PDSA cycles, and track related data.^34^

Between 2015 and 2017, QI collaborative teams from Barbados (1 team), Jamaica (20 teams), Suriname (3 teams), and Trinidad and Tobago (2 teams) tested strategies to improve viral suppression among PLHIV. Viral suppression can occur through daily adherence to antiretroviral therapy and is important for reducing morbidity and mortality as well as reducing the risk of HIV transmission. However, some individuals experience challenges to daily adherence, such as finances and time to collect refills at the pharmacy, medication side effects, and stigma. In each participating country, the MOH supported the collaborative's implementation by sending QI teams to learning sessions and advocating for country-specific, policy-level changes informed by CaReQIC.

A critical aspect of CaReQIC has been engaging PLHIV in the learning sessions and on QI teams. PLHIV were recruited in Jamaica, Barbados, and Trinidad and Tobago via local PLHIV organizations or through facility patient care networks. Suriname entered the collaborative later and therefore their patient engagement experience is not documented here. PLHIV took on this work enthusiastically and requested that they be referred to as community representatives (CRs) to help diminish the power differential in the patient-provider relationship. Table 1 describes CaReQIC's key components.

**TABLE 1.** Key Components of the Caribbean Regional Quality Improvement Collaboration Patient Engagement Strategy

Strategy	Audience/Participants	Purpose	Content for Strategy Success
CaReQIC expert group meetings	I-TECH staff Ministry of Health representatives from each participating country Heads of HIV clinics from Caribbean CRs	Guide the overall technical direction of the collaborative. Ensure CR voices are incorporated at all levels of the collaborative. Help the collaborative maintain a patient-centered focus and culture.	Set global aims. Identify measures. Discuss current issues in each country and priorities across the network. Develop the change package.
Preparatory session for CRs	Patients associated with PLHIV advocacy organizations Key populations, such as MSM, CSWs, and transgender persons, were deliberately included to incorporate the voices of marginalized populations	Introduce CaReQIC and an overview of QI methods, patient engagement, and patient-centered care. Equip CRs with QI skills to participate in technical learning session activities with HCPs. Empower CRs to recognize the importance of their input in all areas of their treatment and care. Prepare CRs to engage in constructive conversations about systems issues in future learning sessions by establishing safe spaces for collaboration.	Discuss QI theory, process maps, swim lane diagrams, test changes (PDSA cycles), identify actionable systems-level issues. Establish learning session expectations, including: role of CRs, creating safe spaces, ground rules of information sharing and confidentiality, avoiding blame, how to give feedback. Explain role of CRs on QI teams.
Patient engagement preparatory session for HCPs	HCPs involved in CaReQIC	Give HCPs an introduction to the patient engagement strategy. Promote the value of patient input and lived experiences. Encourage providers to seek patients' perspectives to improve care. Prepare HCPs to engage in constructive conversations about systems issues in future learning sessions by establishing safe spaces for collaboration.	Describe “patients as partners” in the QI process, benefits of patient engagement, CR roles in learning sessions and QI teams. Establish learning session expectations: creating safe spaces, ground rules of information sharing and confidentiality, avoiding blame, how to give feedback. Encourage “through the patients' eyes”: urge teams to go out and communicate with their patients and see what their experience at the clinic is really like.
CR engagement in learning sessions	CRs and HCPs from facility-based QI teams	Enhance CR and HCP QI technical skills. Improve CRs' and HCPs' ability to work as a team to plan and execute PDSAs at their sites. Encourage perspective-taking and greater empathy between CRs and HCPs.	Highlight coproduction: importance of CR role in QI process, levels of community engagement in QI, co-production of outcomes. Discuss teambuilding and self-identification of strengths. Conduct “learning from the patient experience:” guided conversations between CRs and providers. Hold empathy-building sessions: interactive session to discuss challenges facing HCPs and patients; “problem prism” activity to explore a problem from multiple points of view, including discussion on issues from both patient and provider perspectives; “Ignite Talks” (selected participants share personal stories about experiences as HIV patients or providers working in HIV). Conduct technical QI sessions: setting aims, defining measures, process mapping (including maps guided by CR perspective), swim lane diagrams, run chart development, PDSA planning, change concepts for differentiated care for PLHIV, breakout sessions conducted using ideation technique, "carousel solution” brainstorming to generate a list of change concepts for teams to test for selected drivers, and sustaining changes. Present QI teams' strategies and successes.
CR participation in facility-based QI teams	CRs and HCPs from facility-based QI teams	Provide a mechanism for the PLHIV community, through patient representation, to be actively involved in improving their care and treatment at facilities. Ensure PDSAs aligned with PLHIV priorities.	Team meetings commonly discuss challenges, review patient input, determine strategies, decide which ideas to test, assign tasks, report on progress, complete prework for upcoming learning sessions.
CAST meetings	Led by CRs and I-TECH key population group advisors There were CAST groups in Jamaica and Trinidad and Tobago as of August 2017, with a total membership of 11 CRs	Engage PLHIV to partner with health care facilities to enhance care. Ensure that PLHIV engaged in QI have an opportunity to pass QI skills on to other members of the community. Create a mechanism to recruit new patients, learn QI, and join improvement efforts. Provide a formal conduit to share information and ideas from the PLHIV community to the treatment sites and vice versa. Provide a forum for CRs to troubleshoot challenges they may have with their QI teams.	Conduct monthly meetings, plan and test PDSAs, collect data, present and test change ideas at the site and community level.
Ongoing support to facility-based QI teams	CRs and HCPs from facility-based QI teams I-TECH staff QI coaches	Offer support to QI teams to engage patients and develop and implement new ideas to improve care. Achieve greater patient representation on QI teams. Provide teams with any necessary technical expertise to overcome project hurdles or with engaging patients.	Hold in-person and distance coaching on PDSAs, conduct QI webinars, provide feedback on monthly QI reports, update materials and tools provided, provide data analysis assistance, provide CR recruitment support and related tools provided to QI teams.

Abbreviations: CaReQIC, Caribbean Regional Quality Improvement Collaborative; CAST, CaReQIC Action Strategy Team; CRs, community representatives; CSWs, commercial sex workers; HCPs, health care providers; I-TECH, International Training and Education Center for Health; MSM, men who have sex with men; PDSA, Plan-Do-Study-Act; QI, quality improvement.

The CaReQIC strategy for patient engagement was iterative and adapted to address emergent challenges throughout implementation (Table 1). For example, early in the project, care sites were invited to bring PLHIV with them to learning sessions. Very few did, and some of the PLHIV who came were unclear on their roles and uncomfortable sharing with a room full of strangers. An onboarding approach was needed to ensure that both HCPs and PLHIV were prepared to partner in this work. Based on this need, CR leaders cofacilitated a special session to onboard new CRs before learning sessions.

The CaReQIC strategy for patient engagement was iterative and adapted to address emergent challenges throughout implementation.

Another predicament was related to the targeted recruitment of patients whose needs were not being met by the health care system. To address this challenge, I-TECH reached out directly to organizations that serve the PLHIV community to invite MSM, CSWs, homeless people, and transgender and other marginalized PLHIV to ensure their perspectives were included.

Finally, there was the ongoing challenge of continuity of resources in lower- and middle-income countries to support this type of initiative. Over the years that I-TECH supported this type of work in the region, available funding has waxed and waned. At one point, the project was paused abruptly, requiring the leadership team to accelerate efforts to institutionalize the QI efforts prematurely, only to reinitiate support when funding was restored. This instability in funding was disruptive but was mitigated by I-TECH's deep and ongoing relationships with MOH staff and a strong approach to institutionalization of quality within CaReQIC. While not detailed in this article, I-TECH assisted each health care facility, regional authority, and national MOH to assess their system's ability to support and sustain improvement activities. This resulted in plans to advance the integration of QI across policy, leadership, values, resources, and structures (e.g., staffing, communications, and incentives). During the CaReQIC program's implementation, these needs were addressed directly and indirectly by the teams, the MOHs, and other authorities and through capacity-building activities.

Within this larger context, we focus on the feasibility, acceptability, and perceived value add of CR participation in CaReQIC.

## METHODS

### Data Collection

Between September 2016 and July 2017, qualitative and quantitative data were collected from CaReQIC participants (Table 2), including HCPs and CRs, from Barbados, Jamaica, and Trinidad and Tobago. Two rounds of semistructured, in-depth interviews were conducted. The first round occurred in September 2016. This was approximately 5 months after the first regional learning session and obtained a preliminary sense of the strengths and challenges of the patient engagement strategy. The second round of interviews took place in June and July 2017. Round 2 interview questions were guided by findings from round 1 interview data and focused on how and to what extent CRs have been engaged in QI activities; the perceived value of CR input; and its effect on patient care, systems improvements, and stigma and discrimination. Sampling aimed to capture at least 1 HCP per facility and all participating CRs; however, full coverage was not possible due to respondent availability (Table 1). Purposive sampling was used to select HCPs and CRs with greater exposure to learning sessions and longer experience working with, or as, CRs. Across both sets of interviews, 19 CRs and 22 HCPs were included.

**TABLE 2.** Data Collection Summary in Caribbean Regional Quality Improvement Collaborative Patient Engagement Strategy

Method	Countries	Number of Respondents / Characteristics	Dates
Semistructured interviews with HCPs (via telephone and in-person)	Jamaica	21 respondents from 11 facility-based QI teams 1–4 HCPs/QI team All interviewed QI teams had CRs on their teams	September 2016
	Trinidad and Tobago		
Semistructured interviews with CRs (via telephone)	Jamaica	14 respondents (of 20 CRs total at the time)	September 2016
	Trinidad and Tobago		
Semistructured interviews with HCPs (in-person)	Barbados	5 respondents from 5 facilities	June–July 2017
	Jamaica		
Semistructured interviews with CRs (in-person)	Barbados	9 respondents from 8 facilities	May–July 2017
	Jamaica		
	Trinidad and Tobago		
Community engagement assessment with HCPs and CRs (written questionnaire)	Barbados and Trinidad and Tobago	11 HCPs, 4 CRs	June–July 2017
	Jamaica	42 HCPs, 15 CRs	
	Total: 72 respondents (53 HCPs, 19 CRs) from 23 total facilities		

Abbreviations: CRs, community representatives; HCPs, health care providers; QI, quality improvement.

Written assessment questionnaires, adapted from validated tools used in similar U.S.-based QI projects, were administered to all CR and HCP participants during CaReQIC learning sessions to elicit information on participants' experiences with the patient engagement strategy.^35^ CR- and HCP-specific instruments incorporated a mix of Likert scale responses and open-ended, qualitative feedback. In total, 72 respondents completed the assessments (53 HCPs and 19 CRs).

### Data Analysis

One analyst independently coded interview transcripts and qualitative assessment data, while a second analyst conducted coding validation. Any coding disagreements were resolved through discussion. A hybrid approach of deductive and inductive thematic analysis was used.^36^ Deductive codes were drawn from the literature on patient engagement. Atlas.ti (v.7.5.18, Scientific Software Development GmbH) was used to support coding, analysis, and data organization. Descriptive statistics were generated in Microsoft Excel to analyze the written assessment survey responses and to summarize respondents' sociodemographic data.

### Ethical Considerations

The University of Washington Institutional Review Board determined that the evaluation did not meet the federal definition of human subjects' research, under 45 CFR 46.102 (d) since the intervention and its associated data collection were part of routine program implementation. All participants were at least 18 years of age. Participants gave their verbal informed consent to be interviewed.

## RESULTS

### Collection and Use of Patient Input

Before CaReQIC, HCPs reported little to no systematic gathering or use of patient feedback. Some described informally surveying patients on an ad hoc basis, and a few noted their facilities had suggestion boxes but that they were rarely used. CaReQIC's patient engagement strategy established regular opportunities to learn from CR expertise and for CRs and HCPs to work together on improvement efforts. Most teams met with their CRs at least monthly, though frequency tended to increase before learning sessions to complete preparatory work. Several teams engaged their CRs between scheduled meetings, either in-person, by phone, email, or WhatsApp.

CaReQIC's patient engagement strategy established regular opportunities to learn from CR expertise and for CRs and HCPs to work together on improvement efforts.

All CRs reported participating in developing changes to test with PDSA cycles. Participation included identifying areas for improvement, giving feedback on ideas, assisting with related data collection, and creating storyboards for learning sessions. CRs were also regularly involved as presenters and trainers in learning sessions.

I-TECH supported CRs to develop a group called the CaReQIC Action Strategy Team (CAST), which was formed to activate PLHIV in partnerships with facilities to improve care (Table 1). CAST provides a space for new CRs to learn about QI, for CRs to share and brainstorm together, and for dissemination of successful PDSAs to multiple treatment sites.

### Patient-Provider Partnership as Crucial for Improving Quality of Care

**FIGURE 1:**
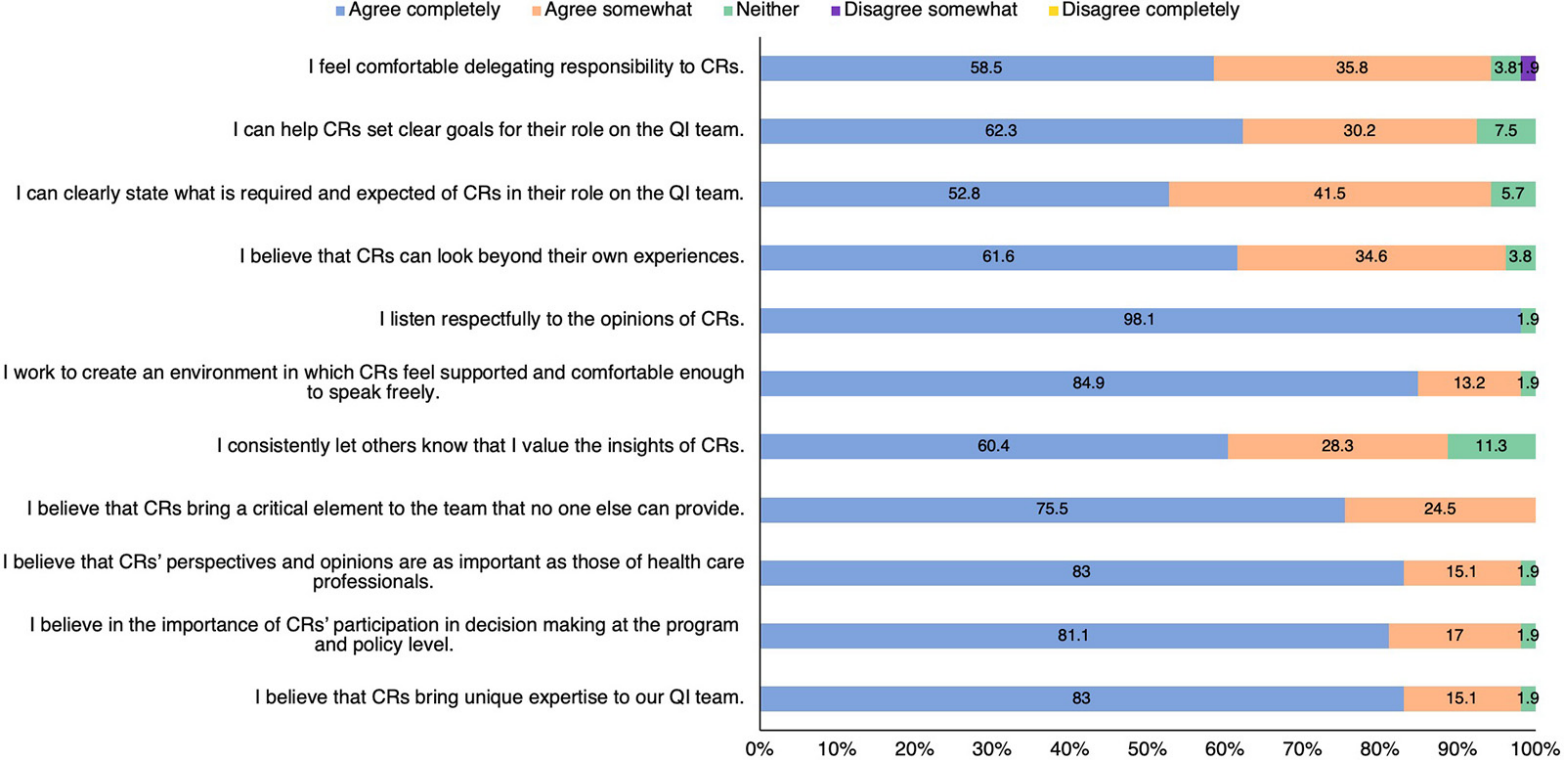
Community Engagement Assessment: Health Care Providers' Responses Abbreviations: CR, community representative; QI, quality improvement.

Across interviews and written assessments, HCPs universally expressed their appreciation for CRs' involvement in the collaborative. Assessment findings found that 98% of providers surveyed either completely or somewhat agreed that CRs' perspectives and opinions were as important as those of HCPs, while 100% completely or somewhat agreed that CRs brought a critical element to the team that no one else could provide (Figure 1). HCPs often suggested CR feedback helped to ensure improvements that effectively catered to client needs, addressed stigma and discrimination, and created a more enabling environment for patients.

HCPs found patient input reminded them of the purpose and importance of their work.

*You get the pulse of the clients. … [I]t is good to get the client's viewpoint because that is the reason for the clinic … you want to provide a service that the client appreciates. Their viewpoint, it helps to keep you grounded and remind you what you are doing … it is good to have that reminder*. —P15, HCP

When asked if facility-based teams planned to continue engaging patients after the collaborative ends, all interviewed HCPs thought they would do so. Several teams had already recruited additional CRs to their teams or were planning to do so at the time of interviews.

### Promotion of CR Empowerment

CRs frequently described how participation in the collaborative made them feel prepared to engage and advocate for improved care for PLHIV. Establishing learning sessions and QI teams as “safe spaces” that valued their unique expertise allowed CRs to feel comfortable sharing their HIV status, ideas, and viewpoints without fear or judgment.

*When I come to this learning session, I say, “Yes, I'm coming to my next family.” … You can talk…about anything related to your status and … you don't feel any discrimination, you just feel light, you just feel welcome, you just feel awesome … . and [I] just feel good about myself.* —P1, CR

CRs also reported their enhanced QI-related skills and knowledge fostered a sense of empowerment. At some sites, CRs helped teach learning session content to other members of their teams who could not attend. CRs explained that gaining technical knowledge and skills gave them confidence, bolstered their roles on QI teams, and enabled them to better contribute to team discussions and PDSA-related activities.

*Participating in QI activities … I feel so empowered now, a part of the whole team. I know I can just go in and give suggestions … Before now, it just felt like they [HCPs] did what they were doing and we came and accessed whatever they did. But now we have our role, we have a say, and they actually listen … and apply other things that we say.* —P26, CR

Leadership and contribution of CRs further grew as the community acknowledged the value of CRs lived experiences and contributions. CaReQIC helped set this tone by conducting sessions on the benefits of CR engagement for improving patient care (Table 1). CRs explained how their participation in CaReQIC made them realize they had a critical perspective to offer that could improve HIV care for themselves and other PLHIV.

*One of the main takeaways is the fact that the community has such a huge say and can be empowered to assist in the changes that impact us. Change in a system cannot be from the top down. It can come from any status, any level, and can be wide reaching—whether doctors or patients—to make change.* —P29, CR

*[CaReQIC] made me realize the value … and the wealth of patient experience and knowledge that I already have from the past that I can bring into CaReQIC … I just want to say thank you … for including us. And when I say us, I mean making the community representatives a part, making our voice known, making our voice heard.* —P8, CR

CRs explained how their participation in CaReQIC made them realize they had a critical perspective to offer that could improve HIV care for themselves and other PLHIV.

**FIGURE 2:**
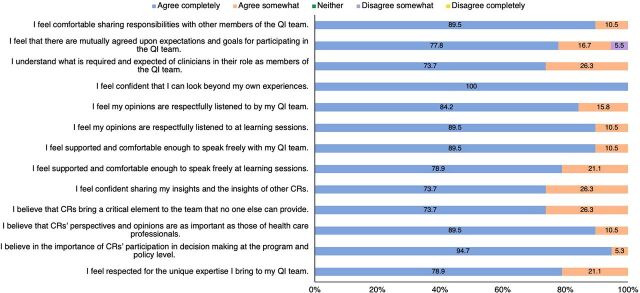
Community Engagement Assessment: Community Representatives' Responses Abbreviations: CR, community representative; QI, quality improvement.

Assessment data compiled in Figure 2 strongly aligned with the qualitative findings that CRs felt welcomed, respected, and supported by other CaReQIC participants. All respondents completely agreed (84.2%) or somewhat agreed (15.8%) that their opinions were respectfully listened to by their QI teams. Similarly, 100% of CRs completely or somewhat agreed that they felt they could speak freely at learning sessions (78.9% and 21.1%, respectively) and with their QI teams (89.5% 10.5%, respectively).

### Enhanced Provider Empathy for HIV Patients Improves Care

Providers frequently expressed how CR involvement in the collaborative learning sessions, particularly the PLHIV testimonies (“Ignite talks”), increased their empathy for patients. Most HCPs found the CR testimonies from vulnerable populations (including MSM, transgender persons, and CSWs) to be eye opening. Increased empathy and awareness were frequently reported to lead to improvements in the care delivered to PLHIV.

*I've been working in this field for many years … but what I find that has helped me in listening to others in the collaborative, and just really tuning in to something that I may have thought I knew or understood, was hearing from community members … Now I'm a little bit more attentive to some of the things that are said, particularly within the MSM population … I know how to better state certain things, how to better ask to get more [information] to be able to make an impact.* —P13, HCP

Many interviewed CRs shared their experiences of improved patient-provider interactions, describing care as “warmer” and “less impersonal.” CRs mentioned improvements like better staff attitude, more 1-on-1 counseling time spent with providers, and feeling like providers were more interested in their perspective.

*I thought it was just great that they [HCPs] would actually listen from the patient's perspective … Because before now, I never got the impression that clinicians were really interested in how we felt … it just felt distant, it felt indifferent. But when they wanted to know how we felt about the care, it made us feel valued. That … you really care that you're doing this to help me … Instead of just doing it because it's your job.* —P6, CR

### Increased CR Empathy for Providers Improves Partnership

CaReQIC also helped CRs to view things from the HCP perspective. This lessened the “us versus them” divide between patients and providers, promoted better communication, and encouraged a stronger sense of teamwork among all participants.

*It was a constant learning experience… . We even shared expectations we had… . It allowed us to actually have an open space to express ourselves freely, in a respectful manner. Because a lot of times we have stigma thinking that these people [HCPs] are against us, you know, and they are just there because they are doing their job. They really want to see persons survive and live as healthy of a life that they can. That was encouraging.* —P28, CR

### Addressing Stigma and Discrimination in Health Care Settings

Learning from PLHIV perspectives, teams implemented several changes aimed at increasing patient confidentiality, comfort, and convenience during their clinic visit. Examples of changes include removing names and identifiers from HIV patient sign-in sheets and extending pharmacy hours. Changes made to reduce patient waiting time had the added benefit of reducing exposure to harassment from other patients.

*I feel good about it, about the extension [of hours]. [Y]ou have some patients who are afraid to come to the clinic for their medication because of people watching them and seeing what they're taking, and so forth. Well in the afternoon hours, not much people are there to scrutinize and to watch who is doing what, you understand?* —P1, CR

Based on PLHIV perspectives, teams made several changes to increase patient confidentiality, comfort, and convenience during their clinic visit.

### Challenges of Moving From Engagement to Partnership

QI teams often sought CR feedback on clinic changes, however, most of the changes that were tested and implemented had been generated by HCPs. Many CRs reported being comfortable with this, as they felt the selected changes aligned with their own priorities, however, some expressed a desire to pursue different ideas and found it difficult to get their teams on board. CRs sometimes found it challenging to advocate for their own ideas because they were the sole PLHIV on the team and the only non-HCP. This amplifies the importance of multiple CRs serving on improvement teams to create strength in numbers. CRs also noted that they faced stronger resistance from HCPs who lacked exposure to learning session content, such as QI methods and the “patients as partners” concept.

Interestingly, some CRs tried to mitigate their team members' resistance to a CR-suggested change by invoking the strengths of QI. They focused on the notion of testing changes on a small scale, with little risk or investment, to convince reluctant team members to test 1 of their ideas.

*So I usually have to say to them, “Remember, it's just a test. We are testing it to see if it works or don't work. If it works, fine.” So sometimes we have to go through all these reminders and go through with the individual, “It's just testing, we're trying to test what I am recommending.”* —P3, CR

There were also some logistical challenges as employed CRs found it difficult to attend QI team meetings during standard work hours and unemployed CRs sometimes lacked transportation to attend meetings. Some teams remedied this by communicating ideas through email and WhatsApp groups. There were strong recommendations from HCPs and CRs alike to remunerate CRs and provide financial support for transportation in addition to the stipend they were provided to participate in learning sessions. It is not only unfair to not compensate CRs for their time but also could amplify inequities based on who can participate and contribute their perspective.

## DISCUSSION

This evaluation found that it is possible to meaningfully engage a marginalized patient population in QI initiatives in lower-resource settings. HCPs reported that PLHIV significantly contributed to the QI process through their suggestions for improving HIV services. Participation of the CRs also renewed HCPs' focus on and commitment to providing excellent patient care through heightened empathy and improved communication skills. CRs reported feeling empowered through participation in the collaborative, their voices were heard, and their experiences valued. CRs and HCPs both described an increase in mutual understanding and intention to sustain partnerships after the collaborative's close. While the countries included in this project are middle-income countries, each has significant inequities in wealth, especially for the marginalized populations of focus in this initiative. The initial challenges and areas of growth shared in the results section are widely applicable to patient engagement strategies in all settings—from lower- to higher-income countries. For example, to build authentic patient engagement, strategies will need to address a wide swath of common challenges from building a culture for improvement (e.g., safe spaces, transparency, and flattened hierarchy) to building shared language and skills for improvement to addressing logistical barriers (e.g., patient transportation, work conflicts, payment and support for expenses like child care). We believe that the learning shared in the results section addresses these common challenges to patient engagement and that these findings are widely applicable and relevant across income levels.

To build authentic patient engagement, strategies need to build a culture of improvement, shared language and skills for improvement, and address logistical barriers.

Our results are well aligned with intergroup contact theory, a well-established theoretical framework that posits that interactions between members of different groups can reduce bias, improve attitudes, and increase acceptance.^37^^,^^38^ Studies have shown the effects of intergroup contact theory are facilitated when 4 optimal conditions are met: equal group status within the intergroup setting, common goals, intergroup cooperation, and authority support.^39^ Here, we describe how QI collaboratives, such as CaReQIC, can create an environment for these optimal conditions to occur among stakeholders in complex systems.

### Equal Status

HCPs do not always recognize the expertise that patients have to offer. QI collaboratives break down the traditional hierarchy of providers over patients, which is important so patients feel more confident speaking to their care team about the opportunities that they see for improvement and so that HCP recognize the need to address patient priorities to achieve improved treatment and care outcomes. Studies have shown that a lack of technical knowledge can prevent patients from participating in improvement activities and that decreasing patient-provider hierarchies is key to achieving meaningful patient involvement.^6^^,^^13^^,^^15^^,^^20^^–^^22^^,^^26^^,^^40^^,^^41^ Several aspects of the CaReQIC intervention served to “level the playing field” and as a result, led to greater patient participation. Providing CRs and HCPs with equivalent QI technical skills and knowledge strengthened their capacity to partner in designing and testing changes. Technical support helps to build patients' skills and confidence, equips them with a “common language” and approach, and makes them feel valued due to a tangible investment in their capacity.^6^^,^^40^ It is likely that including CRs in leadership positions, through learning sessions and participation in the expert group (Table 1), further boosted their visibility and status giving them more opportunities to be meaningfully involved. As has been identified in other studies, our findings suggest hierarchy and power differentials were minimized by increasing participants' awareness of the unique expertise CRs brought to the collaborative.^20^^,^^22^^,^^40^^,^^42^

Efforts to partner with patients are often undermined by assumptions that patients are not experts, lack understanding of the health care system, or lack time or interest to participate in its improvement. These assumptions are reinforced when neither the system nor the efforts to improve it have been designed to partner with the people who live with the condition or illness.^43^ Even the well-intended language of patient engagement can be stigmatizing, implying there is a failure of patients to “engage” in their care. Just as it is important that we use nonstigmatizing language when discussing the impact of HIV on PLHIV, it is also critical that improvement initiatives use language that describes patient partnerships as a productive and equitable alliance, such as coproduction or patient leadership.^44^ QI collaboratives provide a temporary space to demonstrate value and build mindsets which in turn build momentum for a different way of partnering in health systems. It is the responsibility of those designing improvement initiatives and those working in the health care system to remove barriers to patient, family, and caregiver participation in the improvement process. In this project, multiple strategies were tested to remove such barriers and hold space for patient partnership (Table 1).

### Common Goals and Intergroup Cooperation

Improvement initiatives naturally align with intergroup contact theory regarding the need for common goals and intergroup cooperation, as every successful QI initiative defines a common aim and uses a collaborative, multidisciplinary, and structured approach to achieve that aim.^45^ CaReQIC included these core elements and established a shared aim across key stakeholders, including CRs. Learning sessions, webinars, QI coaches, and site visits helped ensure that all participants understood the aim, aligned facility-based improvements toward that aim, and worked together as a team toward contributing goals.

Existing literature helps explain how the methods used in CaReQIC to increase patient-provider cooperation also facilitated patient engagement. The process of preparing health care teams to partner with patients by elevating the expertise that patients hold regarding their own health and their experience navigating the health care system and clarifying their roles on QI teams helped to set the stage for open and productive interactions^20^^,^^40^ Publicly recognizing patient engagement accomplishments built momentum around the partnership across the collaborative and enabled teams to jointly track their progress.^22^ Linking CRs with QI teams from their own facilities allowed CRs to directly observe the outcomes of their involvement and fostered motivation to continue engaging with the collaborative.^22^

Preparing health care teams to partner with patients by elevating the expertise that patients hold regarding their own health and their experience navigating the system helped to set the stage for productive interactions.

### Support of Authorities

Strong leadership support is often cited as a main ingredient to achieving successful patient engagement and commitment to improving the culture of care.^14^^,^^40^ Feedback from CaReQIC participants indicated they perceived a strong degree of endorsement from authorities for the patient engagement strategy, particularly from I-TECH and the MOHs in participating countries. In practice, this was executed by the creation of an expert group to guide the collaborative, comprised of I-TECH staff, MOH representatives, heads of HIV clinics from around the region, and CRs. Involving the MOH in learning sessions was also a way to demonstrate high-level support as well as enhance the likelihood of sustainability.^46^^,^^47^

### Empathy

**FIGURE 3:**
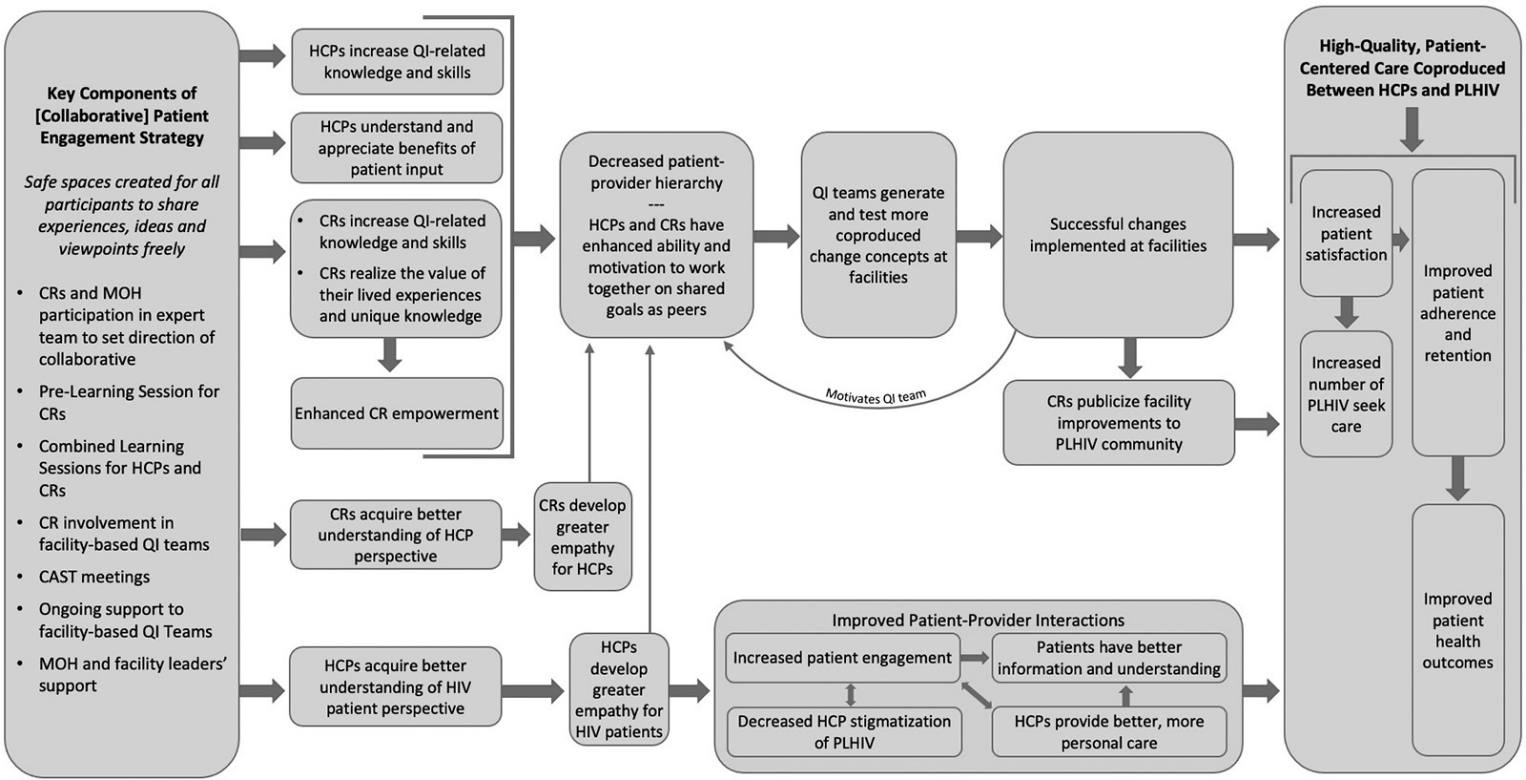
Theory of Change Diagram for Patient Engagement in the Caribbean Regional Quality Improvement Collaborative Abbreviations: CAST, Collaborative Action Strategy Team; CR, community representatives; HCP, health care provider; MOH, Ministry of Health; PLHIV, people living with HIV; QI, quality improvement.

Finally, our study results suggest empathy generated through CaReQIC intergroup activities played a critical role in the intervention's success by fostering improved interactions between CRs and providers and between HCPs and the wider patient population (Figure 3). Several studies have similarly found activities, such as the CaReQIC intergroup activities, generate empathy and in turn reduce stigma.^38^^,^^45^^,^^46^ There is strong evidence that providers' increased empathy and positive attitudes toward CRs would likely be reflected in their interactions with the general patient population as well.^39^^,^^48^^,^^49^

Researchers have linked empathy to increased patient satisfaction,^50^^,^^51^ enhanced willingness by providers to serve PLHIV,^52^ more effective patient-provider communication,^53^^,^^54^ the promotion of patient-centered care,^55^ and improved patient outcomes.^55^ Improved patient-provider interactions are associated with improved HIV-specific outcomes, such as increased patient retention and treatment adherence.^56^^–^^59^

### Strengths and Limitations

As qualitative research, our sampling methods were not intended to yield statistically generalizable findings to other patients or providers. However, our analysis revealed strong thematic saturation and concurrence across the various data sources, suggesting a high level of reliability.^60^ As the focus of our evaluation was on understanding patient and provider experiences with patient engagement, only providers on QI teams with a CR were interviewed. Therefore, we did not capture challenges that may prevent CRs from joining QI teams.

## CONCLUSION

CaReQIC's strategy to integrate patient leadership at every level of the initiative—from improvement teams to leadership structures—offers a promising model to meaningfully involve patients in QI initiatives, especially those involving HIV patients or marginalized patient populations. Consideration of the key conditions and mediators suggested by intergroup contact theory may be particularly useful in designing patient engagement interventions when reaching marginalized populations or when addressing stigmatized illnesses. It is critical to challenge assumptions about patient partnership in health care system improvement and to intentionally create opportunities and spaces for meaningful partnership with patients. A first step in this transformation might be to embrace language that represents the ideal of centering the experience of those impacted by the system and working in true partnership to improve it.

## Notes

**Peer Reviewed**

**First published online:** June 13, 2022.

## References

[1] Institute of Medicine. Best Care at Lower Cost: The Path to Continuously Learning Health Care in America. National Academies Press; 2013. Accessed May 10, 2022. 10.17226/13444 24901184

[2] World Health Organization (WHO). Patient Engagement: Technical Series on Safer Primary Care. WHO; 2016. Accessed May 10, 2022. http://apps.who.int/iris/bitstream/10665/252269/1/9789241511629-eng.pdf

[3] Health Canada and the Public Health Agency of Canada. Guidelines on Public Engagement. Health Canada; 2016. Accessed May 10, 2022. https://www.canada.ca/content/dam/hc-sc/healthy-canadians/migration/publications/health-system-systeme-sante/guidelines-public-engagement-publique-lignes-directrice/alt/pub-eng.pdf

[4] NHS England. Involving People in Their Own Health and Care: Statutory Guidance for Clinical Commissioning Groups and NHS England. Accessed May 10, 2022. https://www.england.nhs.uk/wp-content/uploads/2017/04/ppp-involving-people-health-care-guidance.pdf

[5] Carman KL Dardess P Maurer M. Patient and family engagement: a framework for understanding the elements and developing interventions and policies. Health Aff. 2013;32(2):223–231. 10.1377/hlthaff.2012.1133. 23381514

[6] Ocloo J Matthews R. From tokenism to empowerment: progressing patient and public involvement in healthcare improvement. BMJ Qual Saf. 2016;25(8):626–632. 10.1136/bmjqs-2015-004839. 26993640 PMC4975844

[7] Maclachlan EW Shepard-Perry MG Ingo P. Evaluating the effectiveness of patient education and empowerment to improve patient–provider interactions in antiretroviral therapy clinics in Namibia. AIDS Care. 2016;28(5):620–627. 10.1080/09540121.2015.1124975. 26695005 PMC4841015

[8] Cosgrove DM Fisher M Gabow P. Ten strategies to lower costs, improve quality, and engage patients: the view from leading health system CEOs. Health Aff. 2013;32(2):321–327. 10.1377/hlthaff.2012.1074. 23381525

[9] Coulter A Ellins J. Effectiveness of strategies for informing, educating, and involving patients. BMJ. 2007;335(7609):24–27. 10.1136/bmj.39246.581169.80. 17615222 PMC1910640

[10] Simmons L Wolever RQ Bechard EM Snyderman R. Patient engagement as a risk factor in personalized health care: a systematic review of the literature on chronic disease. Genome Med. 2014;6(2):16. 10.1186/gm533. 24571651 PMC4064309

[11] Hibbard JH Greene J. What the evidence shows about patient activation: better health outcomes and care experiences; fewer data on costs. Health Aff. 2013;32(2):207–214. 10.1377/hlthaff.2012.1061. 23381511

[12] Kim JM Suarez-Cuervo C Berger Z. Evaluation of patient and family engagement strategies to improve medication safety. Patient. 2018;11(2):193–206. 10.1007/s40271-017-0270-8. 28795338

[13] Batalden M Batalden P Margolis P. Coproduction of healthcare service. BMJ Qual Saf. 2016;25(7):509–517. 10.1136/bmjqs-2015-004315. 26376674 PMC4941163

[14] Luxford K Safran DG Delbanco T. Promoting patient-centered care: a qualitative study of facilitators and barriers in healthcare organizations with a reputation for improving the patient experience. Int J Qual Health Care. 2011;23(5):510–515. 10.1093/intqhc/mzr024. 21586433

[15] Han E Scholle SH Morton S Bechtel C Kessler R. Survey shows that fewer than a third of patient-centered medical home practices engage patients in quality improvement. Health Aff. 2013;32(2):368–375. 10.1377/hlthaff.2012.1183. 23381530

[16] Manafò E Petermann L Vandall-Walker V Mason-Lai P. Patient and public engagement in priority setting: a systematic rapid review of the literature. PLoS One. 2018;13(3):e0193579. 10.1371/journal.pone.0193579. 29499043 PMC5834195

[17] Craig GM. Involving users in developing health services. BMJ. 2008;336(7639):286–287. 10.1136/bmj.39462.598750.80. 18230645 PMC2234534

[18] Thomson R Murtagh M Khaw FM. Tensions in public health policy: patient engagement, evidence-based public health and health inequalities. Qual Saf Health Care. 2005;14(6):398–400. 10.1136/qshc.2005.014175. 16326781 PMC1744099

[19] Snow ME Tweedie K Pederson A. Heard and valued: the development of a model to meaningfully engage marginalized populations in health services planning. BMC Health Serv Res. 2018;18(1):181. 10.1186/s12913-018-2969-1. 29544486 PMC5856315

[20] Armstrong N Herbert G Aveling EL Dixon-Woods M Martin G. Optimizing patient involvement in quality improvement. Health Expect. 2013;16(3):e36–e47. 10.1111/hex.12039. 23374430 PMC3883095

[21] Caplan W Davis S Kraft S. Engaging patients at the front lines of primary care redesign: operational lessons for an effective program. Jt Comm J Qual Patient Saf. 2014;40(12):533–540. 10.1016/S1553-7250(14)40069-2. 26111378 PMC4484890

[22] Khodyakov D Stockdale SE Smith N Booth M Altman L Rubenstein LV. Patient engagement in the process of planning and designing outpatient care improvements at the Veterans Administration Health-care System: findings from an online expert panel. Health Expect. 2017;20(1):130–145. 10.1111/hex.12444. 26914249 PMC5217877

[23] Batalden PB Davidoff F. What is “quality improvement” and how can it transform healthcare? Qual Saf Health Care. 2007;16(1):2–3. 10.1136/qshc.2006.022046. 17301192 PMC2464920

[24] Sifrim ZK Barker PM Mate KS. What gets published: the characteristics of quality improvement research articles from low- and middle-income countries. BMJ Qual Saf. 2012;21(5):423–431. 10.1136/bmjqs-2011-000445. 22447823

[25] Franco LM Marquez L Ethier K Balsara Z Isenhower W. Results of Collaborative Improvement: Effects on Health Outcomes and Compliance with Evidence-Based Standards in 27 Applications in 12 Countries. University Research Co.; 2009. Accessed May 10, 2022. https://pdf.usaid.gov/pdf_docs/Pdacr710.pdf

[26] van C McInerney P Cooke R. Patients' involvement in improvement initiatives: a qualitative systematic review. JBI Database Syst Rev Implement Reports. 2015;13(10):232–290. 10.11124/jbisrir-2015-1452. 26571293

[27] The Breakthrough Series: IHI's Collaborative Model for Achieving Breakthrough Improvement. Institute for Healthcare Improvement; 2003. Accessed May 10, 2022. http://www.ihi.org/resources/Pages/IHIWhitePapers/TheBreakthroughSeriesIHIsCollaborativeModelforAchievingBreakthroughImprovement.aspx

[28] Rutledge SE Abell N Padmore J McCann TJ. AIDS stigma in health services in the Eastern Caribbean. Sociol Health Illn. 2009;31(1):17–34. 10.1111/j.1467-9566.2008.01133.x. 18983418

[29] Figueroa JP. Review of HIV in the Caribbean: significant progress and outstanding challenges. Curr HIV/AIDS Rep. 2014;11(2):158–167. 10.1007/s11904-014-0199-7. 24623473

[30] Joint United Nations HIV/AIDS Programme (UNAIDS). The People Living With HIV Stigma Index: Jamaica. An Analytical Report Based on Research Findings. UNAIDS; 2013. Accessed May 10, 2022. http://moh.gov.jm/wp-content/uploads/2016/05/Jamaica-PLHIV-Stigma-Index-Study-Updated-Version-March-9-2015-FINAL.pdf

[31] Rogers SJ Tureski K Cushnie A Brown A Bailey A Palmer Q. Layered stigma among health-care and social service providers toward key affected populations in Jamaica and The Bahamas. AIDS Care. 2014;26(5):538–546. 10.1080/09540121.2013.844762. 24125067

[32] Pan American Health Organization (PAHO). Improving Access of Key Populations to Comprehensive HIV Health Services: Towards a Caribbean Consensus. PAHO; 2011. Accessed May 10, 2022. http://www.paho.org/hq/index.php?option=com_docman&task=doc_view&gid=23834&Itemid=270

[33] Figueroa JP Weir SS Jones-Cooper C. High HIV prevalence among men who have sex with men in Jamaica is associated with social vulnerability and other sexually transmitted infections. West Indian Med J. 2013;62(4):286–291. 10.7727/wimj.2011.207. 24756602 PMC4000540

[34] PDSA cycle. The W. Edwards Deming Institute. Accessed May 10, 2022. https://deming.org/explore/p-d-s-a

[35] National Institute for Children's Health Quality (NICHQ). Family Engagement Guide: The Role of Family Health Partners in Quality Improvement Within a Pediatric Medical Home. NICHQ; 2013. Accessed May 10, 2022. https://www.nichq.org/resource/family-engagement-guide-role-family-health-partners-quality-improvement-within-pediatric

[36] Patton MQ. Qualitative Research and Evaluation Methods. Sage Publications; 2002.

[37] Allportm G. The Nature of Prejudice. Doubleday & Company, Inc.; 1954.

[38] Pettigrew TF Tropp LR. A meta-analytic test of intergroup contact theory. J Pers Soc Psychol. 2006;90(5):751–783. 10.1037/0022-3514.90.5.751. 16737372

[39] Pettigrew TF Tropp LR Wagner U Christ O. Recent advances in intergroup contact theory. Int J Intercultural Relations. 2011;35(3):271–280. 10.1016/j.ijintrel.2011.03.001

[40] Baker GR Fancott C Judd M O'Connor P. Expanding patient engagement in quality improvement and health system redesign. Healthc Manage Forum. 2016;29(5):176–182. 10.1177/0840470416645601. 27576853

[41] Fudge N Wolfe CDA McKevitt C. Assessing the promise of user involvement in health service development: ethnographic study. BMJ. 2008;336(7639):313–317. 10.1136/bmj.39456.552257.BE. 18230646 PMC2234509

[42] Pomey MP Hihat H Khalifa M Lebel P Néron A Dumez V. Patient partnership in quality improvement of healthcare services: patients' inputs and challenges faced. Patient Exp J. 2015;2(1):29–42. 10.35680/2372-0247.1064

[43] Turakhia P Combs B. Using principles of co-production to improve patient care and enhance value. AMA J Ethics. 2017;19(11):1125–1131. 10.1001/journalofethics.2017.19.11.pfor1-1711. 29168684

[44] Lynn V. Why language matters: facing HIV stigma in our own words. The Well Project. Accessed May 10, 2022. https://www.thewellproject.org/hiv-information/why-language-matters-facing-hiv-stigma-our-own-words

[45] Langley G Moen R Nolan K Provost L. The Improvement Guide: A Practical Approach to Enhancing Organizational Performance. Jossey-Bass Publishers; 2009.

[46] Shaller D Darby C. High Performing Patient and Family Academic Medical Centers: Cross Site Summaries of Six Case Studies. Picker Institute; 2009.

[47] Maher LM Hayward B Hayward P Walsh C. Increasing sustainability in co-design projects: a qualitative evaluation of a co-design programme in New Zealand. Patient Exp J. 2017;4(2):44–52. 10.35680/2372-0247.1150

[48] Batson C Polycarpou M Harmon-Jones E. Empathy and attitudes: can feeling for a member of a stigmatized group improve feelings toward the group? J Pers Soc Psychol. 1997;72:105–18. 10.1037/0022-3514.72.1.105. 9008376

[49] Galinsky A Moskowitz G. Perspective-taking: decreasing stereotype expression, stereotype accessibility, and in-group favoritism. J Pers Soc Psychol. 2000;78:708–24. 10.1037/0022-3514.78.4.708. 10794375

[50] Blatt B LeLacheur SF Galinsky AD Simmens SJ Greenberg L. Does perspective-taking increase patient satisfaction in medical encounters? Acad Med. 2010;85(9):1445–1452. 10.1097/ACM.0b013e3181eae5ec. 20736672

[51] Birhanu Z Assefa T Woldie M Morankar S. Determinants of satisfaction with health care provider interactions at health centres in central Ethiopia: a cross sectional study. BMC Health Services Research. 2010;10(1):78. 10.1186/1472-6963-10-78. 20334649 PMC2848139

[52] Lin C Li L Wan D Wu Z Yan Z. Empathy and avoidance in treating patients living with HIV/AIDS (PLWHA) among service providers in China. AIDS Care. 2012;24(11):1341–1348. 10.1080/09540121.2011.648602. 22292939 PMC3419277

[53] Beach MC. Enhancing communications for better patient outcomes. Johns Hopkins Advanced Studies in Medicine. 2010; 10(2):49–52.

[54] Lorié Á Reinero DA Phillips M Zhang L Riess H. Culture and nonverbal expressions of empathy in clinical settings: a systematic review. Patient Educ Couns. 2017;100(3):411–424. 10.1016/j.pec.2016.09.018. 27693082

[55] Lewin SA Skea ZC Entwistle V Zwarenstein M Dick J. Interventions for providers to promote a patient-centred approach in clinical consultations. Cochrane Database Syst Rev. 2001;(4):CD003267. 10.1002/14651858.cd003267. 11687181

[56] Sanjobo N Frich JC Fretheim A. Barriers and facilitators to patients' adherence to antiretroviral treatment in Zambia: a qualitative study. SAHARA J. 2008;5(3):136–43. 10.1080/17290376.2008.9724912. 18979047

[57] Wachira J Middlestadt S Reece M Peng CYJ Braitstein P. Physician communication behaviors from the perspective of adult HIV patients in Kenya. Int J Qual Health Care. 2014;26(2):190–197. 10.1093/intqhc/mzu004. 24519123

[58] Flickinger TE Saha S Moore RD Beach MC. Higher quality communication and relationships are associated with improved patient engagement in HIV care. J Acquir Immune Defic Syndr. 2013;63(3):362–366. 10.1097/QAI.0b013e318295b86a. 23591637 PMC3752691

[59] Flickinger TE Saha S Roter D. Clinician empathy is associated with differences in patient–clinician communication behaviors and higher medication self-efficacy in HIV care. Patient Educ Couns. 2016;99(2):220–226. 10.1016/j.pec.2015.09.001. 26395313 PMC5610904

[60] Sandelowski M. Sample size in qualitative research. Res Nurs Health. 1995;18(2):179–183. 10.1002/nur.4770180211. 7899572

